# The Predictors of Sexual Satisfaction among Iranian Women with Breast Cancer

**DOI:** 10.31557/APJCP.2021.22.2.391

**Published:** 2021-02

**Authors:** Nasrin Fouladi, Iraj Feizi, Mehriar Nadermohammadi, Elham Mehrara, Rozita Adldoosti, Sara Alimohammadi

**Affiliations:** 1 *Department of Community Medicine, Ardabil University of Medical Science, Ardabil, Iran.*; 2 *Social Determinants of Health Research Center, Ardabil University of Medical Sciences, Ardabil, Iran *; 3 *Department of Surgery, Faculty of Medicine, Ardabil University of Medical Sciences, Ardebil, Iran. *; 4 *Department of Psychology, Faculty of Medicine, Ardabil University of Medical Sciences, Ardebil, Iran. *; 5 *Tabriz University, Tabriz, Iran. *; 6 *Faculty of Medicine, Ardabil University of Medical Sciences, Ardebil, Iran. *; 7 *Faculty of Medicine, Shahidbeheshti University of Medical Sciences, Tehran, Iran.*

**Keywords:** Breast cancer, sexual satisfaction, sexual function

## Abstract

**Background:**

Breast cancer targets women’s sexual organs and deals with patients’ femininity. The low age of incidence and the late stage of diagnosis of the disease in Iran give rise to sexual dysfunction among patients. Identifying the severity of the disorder, and its determiners can specify the probable groups to be influenced.

**Materials and Methods:**

In a descriptive cross-sectional study, 144 women with breast cancer who underwent surgical and complementary therapies were included in the study. Data collection was done through questionnaires: FSFI, SSSW and the demographic and clinical information questionnaire.

**Results:**

The mean age of patients was 42.31 ± 5.18 years. 76 patients (52.8%) underwent partial mastectomy and complementary treatments, and 68 cases (47.2%) underwent total mastectomy and complementary treatments. All patients had sexual dysfunction in all dimensions. The average score of sexual satisfaction was 84.3±10 10. The lowest sexual satisfaction score (79.6 ± 9.6) belonged to patients with total mastectomy (P = 0.013). Regression analysis showed predictability of patients’ sexual satisfaction by type of treatment and sexual function (P = 0.002 and P = 0.003, respectively).

**Conclusion:**

Sexual dysfunction and the low level of sexual satisfaction in patients with significant predictive effect of treatment type and sexual function denote that the patients with breast cancer need to be assisted to have proper sexual function and satisfaction leading to higher quality of life.

## Introduction

Breast cancer as the most occurring cancer in Iran accounts for 24.5% of all cancers in women with a standardized incidence age of 28.1 (Salek et al., 2016). The incidence of this disease in Iran is one decade lower than developed countries (Harirchi et al., 2012). Most patients are in the age range of 35- 44 years (Esmkhani et al., 2017).

It affects the sexual organs of women and is associated with the feminine identity of the patients and can negatively affect the sexual relationship of the patients (Landmark and Wahl, 2002). Its treatment also makes patients encounter many sexual problems (Kingsberg., 2010; Rashidi and Dashti., 2015; Oberguggenberger et al., 2017; Fouladi et al., 2018; Fatehi et al., 2019). Breast surgery directly affects the body’s anatomy in women. Changes as a result of radiotherapy entails the unpleasant appearance of breast (Smigal et al., 2006), and breast loss after treatment also damages one’s sexual feelings and causes lack of sexual appeal and exerts negative impact on one’s body-image and ultimately leads to many psychosocial problems in women (Smigal et al., 2006; Fouladi et al., 2013; Fouladi et al., 2018). Chemotherapy also with its subsequent complications disrupts women’s sexual function (Sbitti et al., 2011). Additionally, hormone therapy-induced menopause results in more severe symptoms than normal menopause and reduces libido and fertility and impairs sexual function (Harirchi et al., 2012).

All of these problems affect people’s sexual life (Alacacioglu et al., 2014) and lead to impaired sexual function and a decrease in patients’ quality of life (Fatehi et al., 2019). The prevalence of sexual disorders ranges from 23% to 85% in these patients (Oberguggenberger et al., 2017). Of sexually active patients, 53.2% reported a drop in the amount of sexual relationship after the disease (Leila et al., 2016). Fahami et al., (2015) as well, has reported that 47% of Iranian women with breast cancer have sexual dysfunction. This disease, furthermore, makes women face marital disorders which are related to sexual issues (Leila et al., 2016).

Therefore, breast cancer and its treatment can develop sexual problems both physically and psychologically by the changes that they make (Kingsberg., 2010). But most patients are reluctant to seek help for their sexual problems (Wang et al., 2013), because in most societies, including Iran, talking about sexual dysfunction is taboo (Sbitti et al., 2011). However, this disorder has a negative effect on quality of life and can lead to divorce in families (Safarinejad .,2006).

Most breast cancer management approaches are focused solely on treatment of the disease itself and have turned a blind eye to the psychologic issues associated with the disease and its treatment, particularly sexual ones (Faubion and Kingsberg., 2019). Few research studies have been done on the sexual problems of these patients (Oberguggenberger et al., 2017).

Sexual satisfaction and the factors that influence it are important issues in these patients that require to be investigated. Sexual satisfaction refers to a person’s pleasant feeling of sexual relationship (Saminfar and Vaziri., 2019) and is one of the sources providing many of the psychosocial needs of the couple and is a prominent indicator for judging the effectiveness of a marital relationship. It has a key role in marital satisfaction. Moreover, sexual relationship between couples is dependent on it. Sexuality is also of particular importance in the family domain and numerous studies have reported the impact and importance of sexual satisfaction on family quality and sustainability of marital ties (Saminfar and Vaziri., 2019).

Due to the low age and high rate of sexually active diseases in Iran (Mousavi et al., 2007; Harirchi et al., 2012) and the increased survival rate in these patients owing to different medical treatments, a high proportion of these patients may be exposed to a wide range of sexual problems and require multidisciplinary care (de Morais et al., 2016; Fouladi et al., 2018). Determining the extent of patients’ sexual satisfaction, its severity, and factors affecting these disorders can help identify more vulnerable groups for interventions and can be used in designing interventions and counseling measures for these patients.

## Materials and Methods

This cross-sectional study was conducted in 2018. Project authorization no. (R.ARUMS.REC.2019.098) was obtained from the Ethics and Research of Committee of IR.ARUMS University. 

Using purposeful sampling and taking the inclusion criteria into account, the participants of the study were selected from the population of the patients enrolled in the Cancer Registry Center. The selected patients were called and invited to the closest health care center or hospital to their residential areas. In case of not meeting exclusion criteria and having consent for participating in the study, they were included in the study and were inquired.

The number of women with breast cancer being included in this study was 144, who had undergone surgical and complementary therapies. The inclusion criteria were: being married, not having menopause, not being in control of the disease, and elapsing at least 6 months after the end of treatment and being under 50 years of age, and patients’ consent to participate in the study. Exclusion criteria were having a history of sexual dysfunction before illness, formal sex education over the last month, other serious malignancies or serious illnesses like heart disease, diabetes, asthma, mental illness, such as depression, and consuming anti-Psychotic drugs such as imipramine, diazepam and chlordiazepoxide, genital diseases such as myoma and ovarian cysts and pelvic inflammatory disease, and using drugs and alcohol.

Verbal consent was received from patients and they were allowed to withdraw from the study at any time they wish. The questionnaires were completed in an appropriate and calm environment and without any confounding factors. To collect data, three questionnaires including FSFI, Sexual Satisfaction Questionnaire (SSSW) and Demographic and Clinical Patient Information Questionnaire were employed.

Meston’s Sexual Satisfaction scale for women (SSSW), which measures sexual satisfaction in 5 dimensions, has been validated by Movahed and Azizi in Iran and its reliability has been estimated 95% by Rahimi (Movahed and Azizi., 2011). This self-report scale included 30 questions concerning five domains including contentment, communication, compatibility, concern-relational, concern-personal. The scoring was based on a 5-point Likert scale from strongly agree to strongly disagree. The total score of the scale is calculated by using the following formula: Contentment + Communication + Compatibility + (Relational concern + Personal concern/2). The lowest and the highest scores for the scale are 30 and 150 respectively and there is no cut-off point. Higher scores indicate better sexual satisfaction(Meston and Trapnell., 2005).

Scores for each option ranged from one to five, with the maximum score for each area ranging from 6 to 30. Getting higher score in this questionnaire meant greater sexual satisfaction.

The 19-item Female Sexual Function Index (FSFI) also measures sexual function in six domains of desire, arousal, lubrication, pain, orgasm and satisfaction and its reliability and validity have been studied in Mohammadi’s study in Iranian society (Mohammadi et al., 2008). It was used to collect information on sexual function. Each domain was scored as a 5-point Likert scale for each item, and the cut-off point for the entire questionnaire was 28. The cut off score was considered 3.3 in sexual desire, 3.4 in mental stimulation, 3.4 in lubrication, 3.4 in orgasm, 3.8 in satisfaction and 3.8 in pain..

The demographic questionnaire and clinical characteristics of the patients included data on age and education, occupation, duration of marriage, number of children, type of treatment performed, and time spent completing the treatment.

## Results

The mean age of patients was 31.42 ± 5.18 years, ranging from 29-49 years. The mean duration of marriage was 24.89 years and the highest frequency in terms of education was related to primary and secondary education. Most of the patients, i.e., 100 women (69.4%) were housewives (Table 1).

According to the results of the study, 76 patients (52.8%) had undergone partial mastectomy and complementary treatments and 68 people (47.2%) had undergone total mastectomy and complementary treatments. The majority of patients, 107 cases (74.3%) had spent more than 12 months since completion of their treatment (Table 1). 

The mean score of patients in sexual function was 18.5± 2.3, which was less than the cut-off point (28) indicating that the majority of patients suffered from Sexual Function disorder. Examination of the components of sexual function in patients also showed that the mean scores of patients in dimensions of desire, arousal, lubrication, orgasm, satisfaction, and pain were 3.09 ±0.8, 3.01± 0.48, 2.94 ± 0. 84, 2.91 ± 0.79, 0.74 ± 3.08 , 2.9 0± 0.95, which were lower than cut-off levels in each components of sexual function. 

The measurement of the patients’ satisfaction also showed that the overall mean score of sexual satisfaction was 84.3± 10.

The mean scores of patients in different areas of sexual satisfaction showed that patients’ scores pertaining contentment, communication, compatibility, concern-relational, concern –personal were15.7± 3.6, 17.6 ± 3.2, 17.2 ±3.2, 17.3± 2.9, and 16.3 ± 3.4 respectively. The scores indicated the low scores in each dimension of sexual satisfaction (Table 2).

Patients’ sexual performance was compared considering the type of treatment they received, and the results showed that the sexual function score of patients in all areas of sexual function was lower than the cut-off point regardless of the type of treatment they received. These scores were unrelated to the type of treatment. But there was a significant difference between the type of treatment and patients’ sexual satisfaction (p = 0.013). The group who had undergone partial mastectomy had higher sexual satisfaction scores. And those undergoing total mastectomy with radiotherapy and chemotherapy had the lowest score of sexual satisfaction (Table 3).

In all domains of sexual satisfaction, the lowest score of sexual satisfaction was in patients undergoing total mastectomy. The difference between the component of contentment and the type of treatment and the component of concern-relational and the type of treatment were respectively (p = 0.006, P = 0.018) that were indictor of a significant difference.

There was a significant correlation between the duration of treatment and sexual satisfaction. In other words, those who received more than 12 months of treatment had better sexual satisfaction than those who had completed 6 to 12 months of treatment (p = 0.01, r = 0.21).

The study of the relationship between sexual satisfaction and age of patients also showed that there is an inverse correlation between sexual satisfaction and age (r = -0.22). (p = 0.008), that means sexual satisfaction decreased as the age increased.

There was a decrease in sexual satisfaction among the variables of time spent on marriage with total score of sexual satisfaction (r = -0.23) (p = 0.005).

There was also a significant negative correlation (r = -0.19) between the variable of number of children and the total score of sexual satisfaction, i.e., by the rise of the number of children the sexual satisfaction reduced (P = 0.01). Significant positive correlation was found between sexual function constructs and sexual satisfaction (r = 0.41 (p = 0.00). Regression analysis showed that among all demographic and clinical factors related to patients only sexual function and type of treatment were predictors of sexual satisfaction (Table 4).

**Table 1:** Distribution of Patients' Demographic and Clinical Characteristics

Education Level	
Academic	24 (16%)
Secondary and diploma	30 (20.8%)
high school	53 (36.8%)
Elementary School	37 (25.7%)
number of children	
1	(24.3%) 35
2	(22.9%) 33
3	(38.2%) 55
4 and more	(11.1%) 16
Duration of marriage	5.8±24
Occupation of patients	
Self-employed	33 (22.9%)
Employee	11 (7.6%)
Housewife	100 (69.4%)
Income level	
Sufficient	36 (25%)
Nonsufficient	108 (75%)
Type of treatment	
mastectomy Partial + radiotherapy	38 (26.4)
Partial mastectomy + radiotherapy + chemotherapy	38 (26.4)
Total mastectomy + chemotherapy + radiotherapy	34 (23.6%)
Total mastectomy + chemotherapy	34 (23.6%)
Duration of treatment completion	
Between 6 and 12 months	37 (25.7%)
Over 12 months	107 (74.3%)

**Table 2:** The Women’s Scores for FSFI, SSS-W and Their Subscales

Sexual function domains	mean±SD	Min	Max
Sexual Desire	3.09±0.8	1.2	6
Arousal	3.01±0.48	0	5.7
Lubrication	2.94±0.84	0	5
Orgasm	2.91±0.79	0	5
Satisfaction	3.08±0.74	0.4	5
Pain	2.9±0.95	1	5
Total Score	18.5±1.8	14.6	25.1
Sexual Satisfaction domains			
Contentment	15.7±3.6	9	27
Communication	17.6±3.2	10	25
Compatibility	17.2±3.2	9	26
Relationship concern	17.38±2.9	9	25
Personal concern	16.3±3.4	8	26
Total Score	84±10	59	120

**Table 3:** Mean Scores of Patients' Sexual Satisfaction and Function According to the Type of Treatment

Treatment type	Sexual satisfaction	P value	Sexual function	P value
Partial mastectomy+ Radiotherapy	87.6±10		18.7±1.9	
Partial mastectomy+ Radiotherapy+ Chemotherapy	85±10		16.9±1.8	
Total mastectomy + chemotherapy + radiotherapy	79.6±9.6		17.9 ± 1.4	
Total mastectomy + chemotherapy	83.7±11	0.013	18.2±1.8	0.08

**Table 4:** Regression Analysis for Determining Predictive Factors of Sexual Satisfaction in Patients

	B	sig
age	-0.169	0.071
Education	-0.007	0.928
occupation	0.027	0.726
Duration of marriage	-0.073	0.38
Number of children	-0.132	0.079
Level of income	0.118	0.138
Elapsed time after surgery	0.105	0.168
Type of treatment	-0.231	0.003
Sexual function	0.254	0.002

## Discussion

The results of the study showed sexual dysfunction and the low level of sexual satisfaction in patients with breast cancer. The results showed that the type of treatment and the sexual function are important predictors of sexual satisfaction in women with breast cancer.

All patients in this study scored below the cut-off point, i.e., 28, in sexual function. The findings of Harirchi study also reported sexual dysfunction in 84% of patients with breast cancer (Harirchi et al., 2012). Shandiz’s study has similarly reflected that 67% of women with breast cancer had sexual dysfunction. Moreover, painful intercourse and low sexual desire were the most important sexual dysfunction in these patients (2016). In another study, 77% of patients with breast cancer had sexual dysfunction (Khajehaminian et al., 2014). Cancer has the potential to reduce sexual function and in turn the quality of life due to physical exhaustion and impaired body image. Then it is worth to be seriously considered in long-term survivors of breast cancer to improve overall quality of their lives (Zaker et al., 2019). The results of the present study, in line with other studies conducted in other areas of Iranian society, demonstrate that the sexual dysfunction can be considered a major sexual problem among patients with breast cancer.

It was also found in this study that sexual dysfunction is a problem in all patients with any type of surgery and complementary therapies, which is compatible with the findings of Shandiz and Khajehaminian (2016; 2014). The results of the Sbitti’s study similarly showed that mastectomy leads to decreased libido (2011).

Regarding sexual dysfunction in patients with any type of received treatment, it can be stated that breast cancer diagnosis and treatment creates a physiologically and psychologically stressful situation for patients (Alacacioglu et al., 2014). This may affect sexual function. On the other hand, it is likely that the low age of the disease onset in Iran (Mousavi et al., 2007), which mostly entails the involvement of sexually active women and identifying the disease at the advanced stages in the population under study (Harirchi et al., 2012), increase the likelihood of getting more aggressive type of treatment by patients, and in turn lead to the higher incidence rate of sexual dysfunction in these patients. Therefore, it is suggested that programs be designed for early diagnosis of the patients by conducting regular screening in the community so that the rate of more aggressive treatments and the chance of developing sexual disorders in patients may reduce.

While the highest score can be 150, patients’ satisfaction in this study showed a low score of 84.3 ± 10, and the lowest mean score of sexual satisfaction was related to the patients who had undergone total mastectomy. However, there was a significant relationship between the elapsed time following treatment, the age of the patients, and the number of children, and sexual satisfaction, and these findings are in line with the findings of the studies of Kedde (2013), Lee (2013)and Rosenberg(2014) and contrary to the findings of Oberguggenberger( 2017). However, regression analysis of sexual satisfaction and clinical and demographic characteristics of patients showed only predictive role of treatment and sexual function in patients’ sexual satisfaction.

The study of the pertinent literature indicates that one of the important factors in marital satisfaction is sexual satisfaction and sexual relationship and sexual dissatisfaction can even lead to family breakdown (Foroutan and Jadid milani., 2009). Studies have also uncovered the high impact of sexual dissatisfaction as one of the factors contributing to the divorce rate increase in the normal and healthy population of Iranian society (Foroutan and Jadid milani., 2009; Barikani et al., 2012). Therefore, in breast cancer patients this problem can be much more pronounced than in the healthy population, and cause sexual dissatisfaction and divorce. also reported, 60% of patients with breast cancer didn’t have sexual relationship and 20% divorced (Sbitti et al., 2011). Since marital satisfaction is highly influenced by sexual satisfaction (Saminfar and Vaziri 2019), discovering and solving sexual problems can be of high impact in patients’ sexual satisfaction and reduce family conflicts (Rashidi and Dashti 2015).

A review of the literature also reveals that physical and psychological changes as a result of disease or its treatment can affect the libido of patients’ partners (Kingsberg., 2010). Sexual relationship as a mental concept can be further affected by increased intimate partner communication (Fatehi et al., 2019). Moreover, literature review shows that psychological factors are more powerful than biological factors in sexual relationship (Kingsberg., 2010). Based on the results of this study and the previously done studies, it can be said that interventions with the purpose of reducing sexual problems and increasing sexual satisfaction in these patients, only will be more effective when it is based on educational couples and psychologists. Counselors and sex therapists in hospitals need to consult patients and their spouses and not just treat the disease itself, and these patients need multidisciplinary care because these patients have a wide range of symptoms due to the disease itself or its treatment, especially in relation to the mental image of themselves and sexual issues (Denieffe and Gooney., 2011; de Morais et al., 2016).

Unfortunately, due to cultural barriers, sexual disorders are often not reported by patients as the factors affecting their quality of life, which in turn may cause these problems to remain unsolved in these patients. Effective communication with patients by the health care team can be effective in this context, although communication between health care workers and patients is generally poor (de Morais et al., 2016). Therefore, breast cancer patients in Eastern societies are more likely to have sexual problems than Western women (Sbitti et al., 2011).

Therefore, making staff sensitive to this issue is important and the first step to be taken. By establishing a good rapport with patients, their problems can be identified and effective solutions can be achieved, which can ultimately increase the quality of life in patients.

Contrary to the findings of a study on sexual function showing a sexual dysfunction for any treatment in these patients, this study found that the lowest score of sexual satisfaction was related to the patients undergoing total mastectomy in all areas of sexual satisfaction. Perhaps sexual obedience of the patients in this study, which can be derived from the patriarchal structure of society, can justify this finding. In the society under study, according to the patriarchal structure of society, what is expressed by women’s sexual satisfaction is more likely to be the same as sexual satisfaction of the spouse. In other words, the patriarchal structure implies women’s sexual obedience as well. Thus Sexual obedience can affect the level of sexual activity and sexual satisfaction. Given that sexual satisfaction is a mental concept that may be further affected by an increase in the sexual partner’s intimate relationships (Fatehi et al., 2019), following total mastectomy the patients’ partners’ misbehaviors may have reduced sexual satisfaction in women with total mastectomy more than any other type of treatment. On the other hand, cultural and religious issues also play an important role in the sexual life of women in the study community. The community perceives the women’s’ request for sex negatively, and men mostly initiate sex. The men are more important in this relationship, so if the husband is satisfied, the women will have to be satisfied too (Harirchi et al., 2012). Considering the significant effect of total mastectomy on the women’s’ appearance and the husbands’ dissatisfaction with it, the dissatisfaction expressed by women can be a reflection of their husbands’ sexual dissatisfaction. In other words, the more changes in women’s’ appearance following mastectomy, the less sexual satisfaction can be observed in women. It may suggest that the sexual satisfaction that we estimate not be related to the women’s satisfaction themselves, rather their perceived husbands’ sexual satisfaction, which can limit the findings of the present study, so it would be better to measure men’s sexual satisfaction beside measuring women’s sexual satisfaction.

Given that sexual health is an important aspect of quality of life that is not devoted much attention (Lopresti et al., 2018), it is recommended that the patients with breast cancer be assisted in improving sexual function and satisfaction that can result in enhancing their quality of life. 

Consequently, their sexual dissatisfaction and sexual dysfunction can be lowered by counseling interventions that encompass psychological counselling . As the results of other studies show as well, psychological interventions can also increase the quality of life in these patients by gaining proper control over life conditions (Nural et al., 2019). And ultimately, their sexual health and quality of life can be promoted.
